# Forecasting Weekly Influenza Outpatient Visits Using a Two-Dimensional Hierarchical Decision Tree Scheme

**DOI:** 10.3390/ijerph17134743

**Published:** 2020-07-01

**Authors:** Tian-Shyug Lee, I-Fei Chen, Ting-Jen Chang, Chi-Jie Lu

**Affiliations:** 1 Graduate Institute of Business Administration, Fu Jen Catholic University, New Taipei City 242062, Taiwan; 036665@mail.fju.edu.tw; 2 Artificial Intelligence Development Center, Fu Jen Catholic University, New Taipei City 242062, Taiwan; 3 Department of Management Sciences, Tamkang University, New Taipei City 251301, Taiwan; enfa@mail.tku.edu.tw; 4 Department of Information Management, Fu Jen Catholic University, New Taipei City 242062, Taiwan

**Keywords:** public health, influenza outpatient visits, hierarchical structure, forecasting, decision tree

## Abstract

Influenza is a serious public health issue, as it can cause acute suffering and even death, social disruption, and economic loss. Effective forecasting of influenza outpatient visits is beneficial to anticipate and prevent medical resource shortages. This study uses regional data on influenza outpatient visits to propose a two-dimensional hierarchical decision tree scheme for forecasting influenza outpatient visits. The Taiwan weekly influenza outpatient visit data were collected from the national infectious disease statistics system and used for an empirical example. The 788 data points start in the first week of 2005 and end in the second week of 2020. The empirical results revealed that the proposed forecasting scheme outperformed five competing models and was able to forecast one to four weeks of anticipated influenza outpatient visits. The scheme may be an effective and promising alternative for forecasting one to four steps (weeks) ahead of nationwide influenza outpatient visits in Taiwan. Our results also suggest that, for forecasting nationwide influenza outpatient visits in Taiwan, one- and two-time lag information and regional information from the Taipei, North, and South regions are significant.

## 1. Introduction

Influenza, caused by the influenza virus, is a serious public health issue worldwide, as it is an important cause of individual morbidity and mortality in the population, and existing vaccines are not completely protective [1,2,3]. Influenza pandemics do not only cause a sudden increase in deaths, but also social disruption, and economic loss [4,5]. These effects, although in a more local and lower scale, can result from an epidemic episode even affecting a single country or continent. This has a direct impact on the outpatient departments of hospitals through significant overcrowding [6]. Accurate forecasting of influenza outpatient visits is beneficial for the reasonable planning and allocation of healthcare resources to meet medical demands or to anticipate potential shortages in medical resources. 

Many existing studies use mathematical models, statistical methods, and machine learning to forecast influenza outpatient visits. For example, Towers and Chowell [7] use the susceptible-exposed-infectious-recovered mathematical model to estimate the latent period of influenza; Dugas et al. [8] employed a statistical model of generalized linear autoregressive moving average combined with negative binomial distribution and Google flu trends information to predict influenza cases; Nsoesie et al. [9] combined an individual-based mathematical model and optimization approach for influenza prediction; while Osthus et al. [10] used a deterministic mathematical model of the susceptible-infectious-recovered model to forecast seasonal influenza using multiple sources of uncertainty. 

Volkova et al. [11] proposed a machine learning model of long short-term memory (LSTM) trained by combining data of influenza-like illness with social media signals; its performance was better than when only using historical data of influenza-like illness for influenza-like illness prediction. Basile et al. [1] used a functional generalized least squares regression model to predict epidemic intensity levels; Venna et al. [12] utilized a LSTM neural network model to use the spatiotemporal and environmental factors for influenza prediction. Machine learning does not require strong model assumptions and has advantages in that it is able to build a relatively effective forecasting model. It can also explain non-linear relationships and extract features, and is suitable for analyzing complex, high dimensional data [13]. Thus, machine learning has been adapted in this study to develop an influenza outpatient visit forecasting model. 

The centers for disease control and prevention of a country usually have a duty to track influenza activity, and to monitor and collect data on influenza outpatient visits. The national infectious disease statistics system collects the data, which contain both national and regional statistics. The data on influenza outpatient visits therefore have a hierarchical data structure. The hierarchical data structure is different from a single independent autocorrelation time series, as it is a lag series. Individual time series are grouped by their proximity, and aggregated levels are the sum of the corresponding subsets [14,15]. For example, if we have the regional numbers of influenza outpatient visits, the region is probably a city, county, or state, and the aggregated levels mean that the nationwide numbers are the sum of all of the regional numbers. The national time series collection often has some aggregation structure: An obvious example is the national collection of disease statistics, which records the numbers nationally and covers each city or state. This aggregation is not only a consequence of the data collection method but also a requirement for ensuring privacy.

The existing influenza forecasting studies usually use a nationwide lag time series for multivariate data analysis. The nationwide lag time series can carry information or patterns depending merely on time. In order to collect additional information, the researcher introduced other independent time series, such as Google flu trends, Twitter, Wikipedia, meteorological data, metapopulational data, and laboratory data into the model [1,6,11,12,16,17]. These unstructured variables make it difficult to capture interaction information between variables. The structure patterns of hierarchical influenza data are useful for predicting aggregated nationwide values by capturing the data interactions between each region. The existing studies have supported the efficacy of building forecasting model based on hierarchical data structure [18,19]. For example, Collins [20] presented that the proposed hierarchical forecasting model can generate more accurate forecasts. Dunn et al. [21] and Dangerfield and Morris [22] stated that building a forecasting model using hierarchical data from a lower level can generate better performance.

As regions and time lags often provide useful spatial and temporal information for modeling predictions, this study was based on time lags and region information on influenza outpatient visits to propose a two-dimensional hierarchical influenza forecasting model using machine learning methods. Decision tree methods are one of the most important and frequently used machine learning methods because they have the advantage of being easy to understand and to explain the built model, easy to extract hidden patterns of data, and are suitable for processing unrelated features of data. Random forest (RF) and extreme gradient boosting (XGBoost) are two major decision trees as they can measure the importance of variables and handle high dimensional data [23]. Thus, RF and XGBoost are used in the proposed two-dimensional hierarchical influenza outpatient visit forecasting model to provide effective forecasting results. 

In influenza forecasting, the most important forecasting horizon is one to four weeks ahead. Reich et al. [24] indicate one- and two-weeks-ahead forecasting of influenza are referred to as nowcast, which means the short-time forecasting is approximate to the current time. Similarly, three- and four-weeks-ahead forecasting of influenza is called a forecast, which means long-time forecasting or estimates about events in the future. We considered one- to four-weeks-ahead forecasting at the same time to include both views of nowcast and forecast. In this study, we used a multi-stage forecasting strategy to predict influenza outpatient visits one to four steps ahead. To evaluate the reliability of the proposed two-dimensional hierarchical decision tree scheme for influenza outpatient visit forecasting, popular statistical methods for medical data forecasting, including autoregressive integrated moving average (ARIMA) and multivariate adaptive regression splines (MARS), were used in this study for comparison [25,26].

## 2. Methods

### 2.1. Random Forest 

RF is a combination classification method of a supervised machine learning algorithm based on a decision tree, and aims to build up a collection of decision trees [27]. A popular aggregation function is considered as the unweighted majority of class votes. RF changes constructed methods of classification or regression trees. RF uses the best value among a subset of predictors randomly chosen at that node to split each node. In the process, RF uses a different bootstrap sample of the data to construct each tree, and uses the bagging program to select multiple random variable samples as the training data set. The bagging program indicates replacing the meta-algorithm with random sampling. Then, under the training process, a tree classifier corresponding to the selected sample is constructed, and a large number of the trees from the selected samples are used to produce an RF. 

The typically tree-based classifier of RF is a classification and regression tree. RF uses the metric called out of bag error to measure the performance, which is the average of the rate of error in each weak learner. Each tree of the RF depends on the sampling random vector, and all trees in the forest have the same distribution. When the trees are developed to its maximum expansion, RF will use a particular method to explore each individual tree and select the most important variable for splitting randomly [28]. The common stopping criteria of RF for the split are that the number of samples in the leaf node is less than the given threshold, and the Gini index in the sample set is less than the given threshold. 

### 2.2. XGBoost

XGBoost is an advanced method based on the tree boosting algorithm. It can perform massive parallel-boosted tree calculations. It optimizes the loss function of the traditional gradient boosting decision tree model, and provides regularization, a new sparsity-aware algorithm, a distributed weighted quantile sketch algorithm, and optimizes the cut-point search algorithm. The algorithm optimizes the value of the objective function and applies the feature vectors for prediction. The gradient enhancement builds an enhancement tree to adaptively obtain feature scores, indicating the difference in importance of each feature in the training model. The enhanced tree is used for key decision-making, in which some tree features are more, and the score is higher. XGBoost calculates the importance of features by gain, frequency, and coverage [29]. 

The XGBoost algorithm uses the regular term in the cost function to effectively prevent overfitting and to perform a second-order Taylor expansion of the cost function, and uses both first-order and second-order derivatives, while applying the loss function to calculate the pseudo-residuals of the first and second derivatives for generating learning. Its *t*-th loss function can be expressed as [25]:

(1) $${ℒ}^{\left(t\right)}=\overset{n}{\underset{i=1}{\sum }}l\left(y_{i},{\hat{y}}_{i}{}^{\left(t-1\right)}+f_{t}\left(x_{i}\right)\right)+\Omega \left(f_{t}\right)$$

XGBoost considers the inefficiency of the greedy method, so it uses an approximate algorithm to find the best segmentation point. It can use the sparse value to specify the default direction of the branch for a missing value or specified value, improving the efficiency of the algorithm [30]. When the gain of new branches is less than zero or the tree reaches the maximum depth, the XGBoost algorithm will be stopped.

### 2.3. Autoregressive Integrated Moving Average

The autoregressive integrated moving average (ARIMA) is constructed on historical data and attempts to identify patterns in the data. An ARIMA model contains three components: Autoregressive (AR), moving average (MA), and an integrated component. An ARIMA model is a generalization of an ARMA model [31]. In simple terms, the ARIMA model of nonstationary random process can be described as ARIMA (*p,d,q*) by the order of AR, the difference (integrated component), and MA, and its mathematical model can be denoted as [32]:

(2) $$\left(1-\overset{p}{\underset{i=1}{\sum }}\alpha_{i}B^{i}\right){\left(1-B\right)}^{d}Y\left(t\right)=\beta_{0}+\left(1-\overset{q}{\underset{i=1}{\sum }}\beta_{i}B^{i}\right)\varepsilon \left(t\right)$$

where *B* is the backshift operator; {$\alpha_{i}$} are the AR coefficients; {$\beta_{i}$} are the MA coefficients; ε(t) are the white Gaussian noise process with mean zero and variance $\sigma^{2}$; $\beta_{0}$ is the deterministic trend term, and there is an implied polynomial of order *d* in the forecast function when d> 0. Therefore, $\left(1-\sum_{i=1}^{p}\alpha_{i}B^{i}\right)$ and $\left(1-\sum_{i=1}^{q}\beta_{i}B^{i}\right)$ are polynomials of order *p* and *q*, respectively. The Box-Jenkins methods of model identification for a non-stationary time series can be abstracted in four steps [33]. The first step is selecting the appropriate conversion of the observed time series. The most basic transformations are variance-stable transformations and differential operations. The second step is calculating the ACF (Auto-correlation) and PACF (Partial Auto-correlation Function) of the samples in the observed time series to determine the necessity and degree of difference. In the third step, we can identify the orders of *p* and *q* for the ARIMA model depending on calculating the sample ACF and PACF of the correctly transformed time series. In the last, if *d* > 0, we test the deterministic trend term $\beta_{0}$ to determine the necessity of including $\beta_{0}$ in the model.

### 2.4. MARS

MARS is a new technology of nonparametric statistics using a multivariable nonlinear algorithm to aim at finding optimal variable transformations and interactions. It is a flexible process to model relationships that are approximately additive or involve interactions with fewer variables. The model is a sum of several basic equations or the basis function (BF), and then MARS combines them for prediction. Under the modeling process, MARS is dependent on a divide-and-conquer strategy. The training data sets are divided into separate regions and each region has its own regression equation. In different intervals of the independent variable space, MARS uses the separate linear regression slopes in distinct intervals to approximate the non-linearity of a model [34]. We can use the following mathematical formula to express it:

(3) $$\hat{f}\left(x\right)=\alpha_{0}\overset{m}{\underset{i=1}{\sum }}\alpha_{i}\overset{kn}{\underset{k=1}{\prod }}\left[p_{kn}\left(x\left(k,\;n\right)-t_{kn}\right)\right]$$

where $\alpha_{0}\;\mathrm{and}\;\alpha_{i}$ are parameters, m is the number of basic functions, $k_{n}$ is the number of knots, $p_{kn}$ is given as values of either 1 or −1, indicating the right/left sense of the associated step function, x(k, n) is the label of the independent variable, and $t_{kn}$ means the knot location. We can consider that $\alpha_{0}$ and $\alpha_{i}$ give a value similar to regression coefficients, mainly the number of BF determined by the evaluation criteria.

We can infer that the data points within the expectation interval show a linear relationship, and then use a linear function (basic equation) in each interval to describe the relationship between the data in the interval. Among the intervals, the node or knot is used as a separation point. This means that these nodes are not only the inflection point where the slope of the equation changes at each interval, but also where the data behavior changes. After accumulating the basic equations of these intervals, we can obtain a more flexible nonlinear model to solve complex problems that are multiple and nonlinear. In other words, there are three steps for MARS procedure. In the first step, the algorithm is employed to pick all probable basic functions and their corresponding knots. The second step is in order to produce the best combination of existing knots. MARS is dependent on the general cross-validation (GCV) criterion using a backward algorithm to delete all basic functions in the order of the least contribution. GCV is a form of regularization, which can balance goodness-of-fit against model complexity. When the lowest GCV value is reached, the best combination is chosen.

### 2.5. Model Implementation 

The methods used in this study have been implemented in R of version 3.61 (R core team, Vienna, Austria). The algorithm of the methods is based on the relevant R package. There are five R packages used in our study; in particular, the *e1071* R package of version 1.7-3 [35] is used to construct the RF model and the *tuneRF* function of *e1071* R package is used to tune and obtain the best model. Then, we implemented MARS using the *earth* R package of version 5.1.2 [36]. The default setting of this package was used to tune and then build the best additive MARS model.

Because we are trying to determine the importance of variables from RF, the *RandomForest* R package of version 4.6-14 [37] was used to extract the important variables from the best model of RF. In the next section, we find the optimal orders for ARIMA using the *auto.arima* function of the *forecast* R package of version 8.11 [38] to provide a method to compute the best orders for the model automatically, and then we use the order to build the ARIMA model. Finally, the XGBoost model was constructed using the *xgboost* version 0.90.0.2 of the R package [39] for tuning and modeling the best XGBoost model. 

### 2.6. Proposed Influenza Outpatient Visit Forecasting Scheme

**Figure 1:**
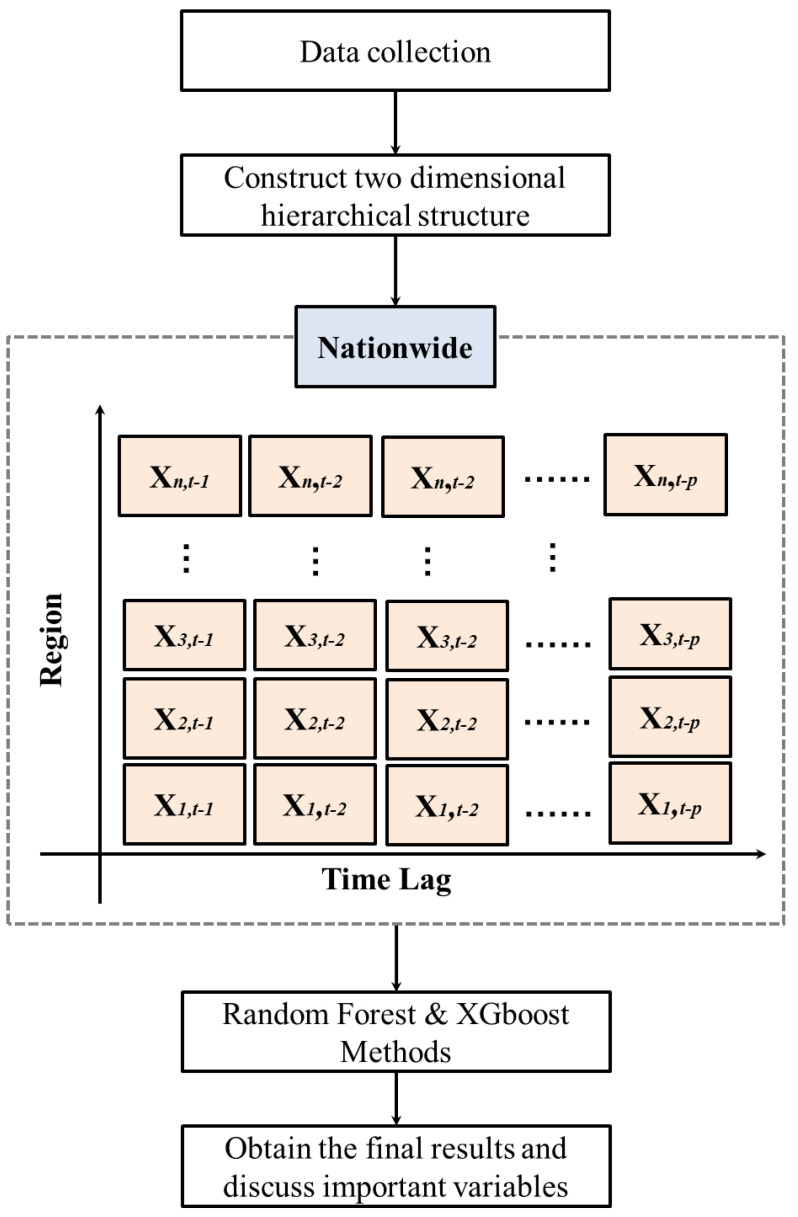
The flowchart of the proposed influenza outpatient visits forecasting scheme.

In this study, two decision tree techniques, RF and XGBoost, were integrated into a two-dimensional hierarchical data scheme to build an effective scheme for predicting influenza outpatient visits in Taiwan. A flowchart of the proposed scheme is shown in Figure 1.

The first step in the proposed scheme was collecting the data. The second step was to construct a two-dimensional hierarchical structure based on nationwide and regional data. The two-dimensional hierarchical structure considered the regional values, and used the time lag of each region to generate potential predictor variables for the RF and XGBoost methods. In this structure, the total number of outpatient visits (nationwide) was equal to the sum of the outpatient visits of all regions, so we were able to obtain a data matrix of size n by p that constructed the number of influenza outpatient visits in n regional and its p time-lag. That is, the number of influenza outpatient visits in the region n and time-lag p of the two-dimensional hierarchical structure can be expressed as $X_{n,t-p}$. 

For example, as depicted in Figure 1, $X_{1,t-1}$ represents the number of influenza outpatient visits in the region 1 and lag 1 week; $X_{3,t-2}$ stands for the number of influenza outpatient visits in the region 3 and lag 2 weeks, and so on. 

As the Taiwan influenza data contains six regions, namely, the Taipei, North, Central, South, Kaoping, and East regions, n=6 was used in this study. The notations {T,N,C,S,K,E} are, respectively, corresponding to the Taipei, North, Central, South, Kaoping, and East regions. Moreover, since the time-lag information of one to four weeks was the one most used and produced reasonable information for influenza outpatient visit forecasting [24], p=4 was considered in this search. Therefore, we could generate 24 predictor variables, $X_{n,t-p}$, n={T,N,C,S,K,E}, p=1,2,3,4. The target variable was the total (nationwide) influenza outpatient visits at time t ($Y_{t}$). 

In the third step, we used RF and XGBoost decision tree methods to predict nationwide influenza outpatient visits using the generated predictor variables. In the two models constructed, the 24 hierarchical variables were directly used as predictors for RF and XGBoost. The model used RF and was called a two-dimensional hierarchical random forest (TDHS-RF) scheme. Similarly, the model utilizing XGBoost as the prediction method was called TDHS-XGB. Meanwhile, the MARS was also used in the proposed two-dimensional hierarchical scheme to evaluate the advantage of the decision tree, which was termed TDHS-MARS. Moreover, the RF, XGBoost, MARS, and ARIMA methods, without incorporating a two-dimensional hierarchical-based scheme, were used as benchmarking methods for evaluating the proposed influenza outpatient visit prediction scheme.

In order to evaluate the performance of the proposed scheme and the competing methods, five commonly used performance metrics including mean absolute error (MAE), root mean square error (RMSE), mean absolute percentage error (MAPE), mean absolute scaled error (MASE), and root mean square percentage error (RMSPE) were used in this study. The definitions of these metrics are summarized in Table 1. These metrics were used to measure the deviations between the actual and predicted values. The deviation was smaller, and the accuracy was higher.

In the last step, after obtaining the final forecasting results of each model, we compared the prediction errors of all the models and obtained the final results for predicting influenza outpatient visits in Taiwan. The meanings of the identified important variables were also discussed. 

## 3. Empirical Study

**Figure 2:**
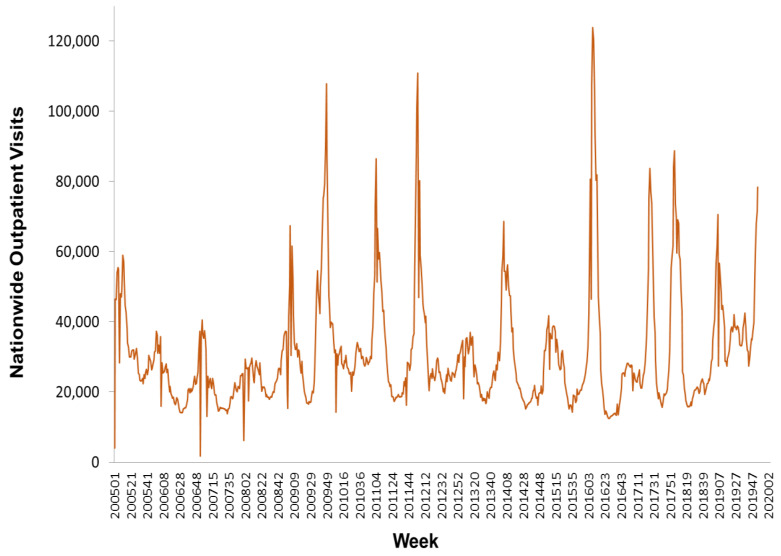
The nationwide outpatient visits for influenza in Taiwan from the first week of 2005 to the second week of 2020.

**Figure 3:**
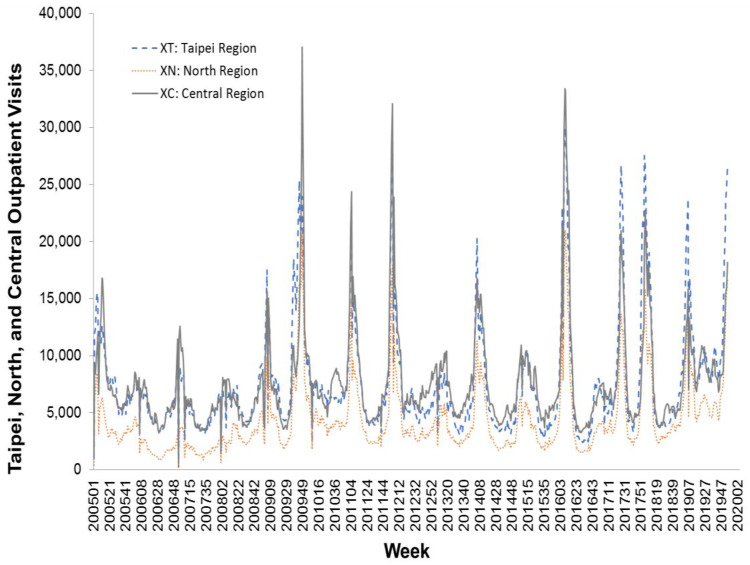
The influenza outpatient visits for Taipei, North, and Central regions in Taiwan from the first week of 2005 to the second week of 2020.

**Figure 4:**
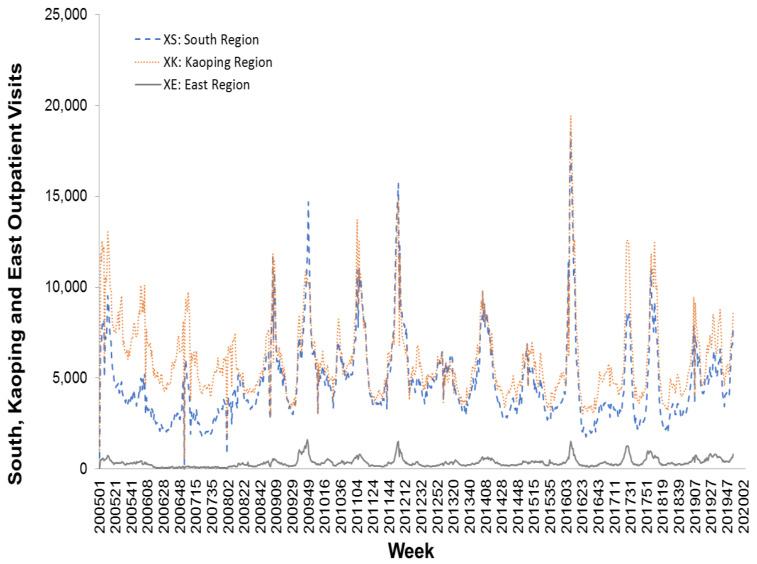
The influenza outpatient visits for South, East, and Kaoping regions in Taiwan from the first week of 2005 to the second week of 2020.

In this study, we used weekly influenza outpatient visit data collected from Taiwan’s national infectious disease statistics system (https://nidss.cdc.gov.tw/en/). The data range was from the first week of 2005 to the second week of 2020, and included 788 data points. The dataset contained nationwide outpatient visits for influenza, and six regional outpatient visits for influenza. The regions were Taipei, North, Central, South, East, and Kaoping, and the nationwide values were the sum of the six regional values. Figure 2, Figure 3 and Figure 4 depict Taiwan’s total national influenza outpatient visits from the first week of 2005 to the second week of 2020.

The sliding window approach was used in this study to split the data into training and testing data sets based on their time period. After data cleansing, the first 630 data points (80% of the total sample points, from the first week of 2005 to the first week of 2017) were selected as the training samples, while the remaining 158 data points (20% of the total sample points, from the second week of 2017 to the second week of 2020) were employed as the testing sample for measuring the out-of-sample forecasting ability of the proposed scheme.

The proposed TDHS-RF and TDHS-XGB models were used in one to four step (week) forecasts of nationwide influenza outpatient visits in Taiwan. First, Table 2 presents the results of one-step (week)-ahead outpatient visit forecasting of the proposed TDHS-RF and TDHS-XGB models, and the competing models including TDHS-MARS, RF, XGBoost, MARS, and ARIMA. As Table 2 depicts, the performance of the proposed TDHS-RF and TDHS-XG models are better than those of the five comparison models, and the TDHS-XG model can provide the best forecasting accuracy of all the models for one-week-ahead nationwide influenza outpatient visit forecasting. It can also be observed from Table 2 that both the TDHS-RF and TDHS-XGB models outperformed the RF and XGBoost models, which did not use two-dimensional hierarchical structure data. This indicates that the proposed data scheme can be used to improve forecasting accuracy. In addition, Table 2 shows that all machine learning-based methods, including TDHS-RF, TDHS-XGB, RF, and XGBoost, outperformed the statistical-based methods, including TDHS-MARS, MARS, and ARIMA.

Table 3 shows the forecasting results of two- to four-steps (weeks)-ahead of outpatient visits of the proposed TDHS-RF and TDHS-XG models, and the five comparison methods. Table 3 shows that the proposed TDHS-RF and TDHS-XGB models outperformed the five comparison methods in all two- to four-step (week) forecasts. The machine learning forecasting models can generate better forecasting results than the statistical methods including ARIMA and MARS. Thus, it can be concluded that the proposed TDHS-RF and TDHS-XG models are effective and promising alternatives for forecasting one to four steps (weeks) ahead for nationwide influenza outpatient visits in Taiwan.

Table 4 shows the relative importance value of each predictor variable in decreasing order based on the importance identification results of the proposed TDHS-RF and TDHS-XGB models. It is the average value of the variable importance values of one- to four-step forecasting using the TDHS-RF and TDHS-XGB models. From Table 4, it can be found that X_T,t−1_ is the most important variable. It means that the number of influenza outpatient visits in the Taipei region and lag one week is the most important reference information to plan and allocate healthcare resources for influenza prevention.

## 4. Discussion

**Figure 5:**
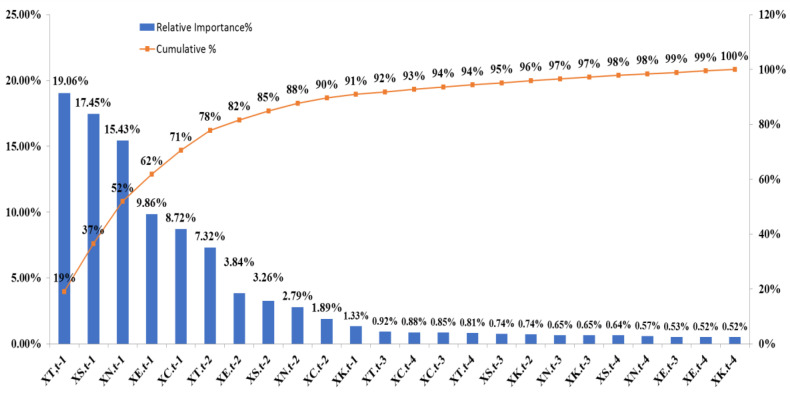
The relative and cumulated importance values of each predictor variables.

Figure 5 shows the relative importance value and cumulative importance value of each variable based on Table 4. For identifying important variables, after discussing with three experts in public health, the first three variables such as X_T,t−1_, X_S,t−1_, and X_N,t−1_ are inferred to be the most important variables and can be considered as a crucial sign for influenza prevention. From Figure 5, we can find the cumulative importance of the first three important variables is 52% which is around 60% and these three variables account for only 12.5% of all 24 variables. According to the first three important variables, we can infer that when the number of influenza outpatient visits in the previous one week are bumping up in the Taipei, South, and North regions, it is highly probable that within the next four weeks, there will be a nationwide pandemic. From an administrative area perspective, it seems reasonable because the Taipei region includes Taipei City, New Taipei City, Keelung City, Yilan County, Kinmen County, and Lianjiang County. The North region includes Hsinchu City, Hsinchu County, Taoyuan City, and Miaoli County; and the South region includes Tainan City, Chiayi City, Chiayi County, and Yunlin County. These regions cover the most important areas of economic activity in Taiwan.

For using more important variables for influenza prevention, the experts suggested that the first ten variables including X_T,t−1_, X_S,t−1_, X_N,t−1_, X_E,t−1_, X_C,t−1_, X_T,t−2_, X_E,t−2_, X_S,t−2_, X_N,t−2_, and X_C,t−2_ could be considered since these then variables account for only 41.6% of all 24 variables. The cumulative importance value the first ten important variables is 90% which is also a common concept in the statistical analysis [40]. The first ten important variables depict the geographical area of Taipei, South, North, Central, and the East regions, and its influenza outpatient visits in the previous one and two weeks are important, and the variables for lag three to four weeks are not selected. The result presents that the high-frequency variable in this study is less of a contribution to predict influenza outpatient visits nationwide. The first ten variables can be used to predict the trend of influenza outpatient visits one week to four weeks ahead. 

## 5. Limitations and Future Research 

This study only used weekly influenza outpatient visits of Taiwan to evaluate the performance of the proposed scheme and to find important variables as a meaningful sign for influenza prevention. The findings of this study could not be directly extended to other countries’ outpatient visits of influenza is one of the limitations. The other limitations of this study are that outpatient visits of influenza-like illness, emergency department visits of influenza, and influenza-like illness were not considered in this study. 

The LSTM, one of the state-of-the-art machine learning methods, is an attractive method and has been used for influenza forecasting study in recent years [11,12]. But, the main disadvantage of LSTM is that it has a complicated training model which can easily get overfitting and require a long training time [41,42]. Meanwhile, LSTM cannot be used to select important predictors. On the contrary, RF and XGboost have the advantages of being easy to build and explain forecasting models, easy to avoid overfitting, and can select important predictors for further analysis. Moreover, RF and XGBoost are both promising methods and have been successfully used for different applications in recent years [43]. Thus, as the main aim of this study was to propose a two-dimensional hierarchical decision tree scheme for forecasting influenza outpatient visits and identifying important predictor variables, using LSTM in the proposed scheme and comparing the performance between the proposed scheme and the LSTM may not useful for our research purpose, although it might be one of our research limitations. 

According to the limitations, using the proposed two-dimensional hierarchical decision tree forecasting scheme for other countries’ influenza outpatient visit data and emergency department visits of influenza could be one of the future research directions. Moreover, as using the state-of-the-art machine learning methods with feature selection mechanism in the proposed scheme might generate a more promising influenza outpatient visit forecasting scheme, it would be considered as one of our future research subjects. In addition, utilizing more hierarchical variables such as population and economy indexes in the proposed scheme to provide a deeper insight for influenza prevention could be also be taken into account for our future study. 

## 6. Conclusions

This study was based on spatial and temporal information on influenza outpatient visits to propose a two-dimensional hierarchical decision tree scheme for forecasting nationwide influenza outpatient visits in Taiwan. In the proposed scheme, first, a data matrix of predictor variables was constructed by the number of influenza outpatient visits in six regions and its four time-lags. The twenty-four predictor variables, including spatial and temporal information, were then used as input variables of RF and XGBboost algorithms to respectively construct the TDHS-RF and TDHS-XGB models. Finally, the proposed TDHS-RF and TDHS-XGB models were utilized to forecast one- to four-steps (weeks)-ahead nationwide influenza outpatient visits in Taiwan and identify important variables. 

The empirical results demonstrated that the proposed TDHS-RF and TDHS-XGB models were effective and promising alternatives for forecasting one to four steps (weeks) ahead for nationwide influenza outpatient visits in Taiwan. Our results also suggest that, for forecasting nationwide influenza outpatient visits in Taiwan, one- and two-time lag information and region information from the Taipei, North, and South regions were most significant. If the numbers of influenza outpatients are significantly increased in those regions, officials should pay attention as there is a high probability of a nationwide influenza pandemic occurring within one to four weeks. This important spatial and temporal information can provide useful information to prepare and design reasonable planning and allocation of healthcare resources to prevent a potential influenza pandemic.

## Figures and Tables

**Table 1:** The equations of the five performance metrics.

Metrics	Calculation *
MAE	$\mathrm{MAE}=\frac{1}{m}\overset{m}{\underset{j=1}{\sum }}\left|F_{j}-A_{j}\right|$
RMSE	$\mathrm{RMSE}=\sqrt{\frac{1}{m}\overset{m}{\underset{j=1}{\sum }}{\left(F_{j}-A_{j}\right)}^{2}}$
MAPE	$\mathrm{MAPE}=\frac{1}{m}\overset{m}{\underset{j=1}{\sum }}\left|\frac{F_{j}-A_{j}}{A_{j}}\right|\times 100$
MASE	$\mathrm{MASE}=\frac{\frac{1}{m}\sum_{j=1}^{m}\left|F_{j}-A_{j}\right|}{\frac{1}{z-1}\sum_{t=2}^{n}\left|A_{t}-A_{t-1}\right|}$
RMSPE	$\mathrm{RMSPE}=\sqrt{\frac{1}{m}\overset{m}{\underset{j=1}{\sum }}{\left(\frac{F_{j}-A_{j}}{A_{j}}\right)}^{2}}$

* Note that *F* and *A* represent the predicted and actual value; m is the total number of testing set; *z* is the total number of training set.

**Table 2:** Forecasting results of one step (week) ahead outpatient visits.

Method	MAE	RMSE	MAPE	MASE	RMSPE
TDHS-RF	4693	8702	0.126	1.167	0.188
TDHS-XG	4409	8050	0.113	1.096	0.168
TDHS-MARS	6415	14,000	0.159	1.595	0.226
RF	5040	9179	0.133	1.253	0.194
XGB	4432	8029	0.117	1.102	0.173
MARS	5158	9861	0.133	1.282	0.201
ARIMA	13,986	20,774	0.417	3.477	0.512

Note that TDHS–RF means two-dimensional hierarchical random forest; TDHS-XGB means two-dimensional hierarchical extreme gradient boosting; TDHS-MARS means two-dimensional hierarchical multivariate adaptive regression splines; RF means random forest; XGB means extreme gradient boosting; MARS means multivariate adaptive regression splines; ARIMA means autoregressive integrated moving average.

**Table 3:** Forecasting results of two to four steps (weeks) ahead of outpatient visits.

Step (Weeks) Ahead	Method	MAE	RMSE	MAPE	MASE	RMSPE
2	TDHS-RF	6451	10,614	0.176	1.599	0.240
	TDHS-XGB	6439	11,018	0.163	1.596	0.225
	TDHS-MARS	8077	14,819	0.205	2.002	0.268
	RF	6858	11,342	0.186	1.700	0.264
	XGB	6489	11,009	0.168	1.608	0.231
	MARS	7071	11,689	0.185	1.753	0.247
	ARIMA	14,037	20,818	0.418	3.479	0.513
3	TDHS-RF	8222	13,098	0.227	2.033	0.316
	TDHS-XGB	8329	13,764	0.211	2.059	0.291
	TDHS-MARS	9671	16,012	0.252	2.391	0.328
	RF	8691	13,857	0.241	2.149	0.346
	XGBoost	8564	14,119	0.222	2.118	0.312
	MARS	8780	14,054	0.234	2.171	0.323
	ARIMA	14,082	20,862	0.419	3.482	0.514
4	TDHS-RF	9994	15,609	0.280	2.463	0.401
	TDHS-XGB	10,178	16,550	0.264	2.508	0.375
	TDHS-MARS	11,114	17,746	0.295	2.739	0.404
	RF	10,478	16,595	0.295	2.582	0.435
	XGB	10,433	17,027	0.274	2.571	0.401
	MARS	10,462	16,654	0.285	2.578	0.414
	ARIMA	14,118	20,903	0.419	3.479	0.514

**Table 4:** The relative importance value of each predictor variable based on the proposed schemes.

Variable	Relative Importance	Variable	Relative Importance
X_T,t−1_	19.06%	X_C,t−4_	0.88%
X_S,t−1_	17.45%	X_C,t−3_	0.85%
X_N,t−1_	15.43%	X_T,t−4_	0.81%
X_E,t−1_	9.86%	X_S,t−3_	0.74%
X_C,t−1_	8.72%	X_K,t−2_	0.74%
X_T,t−2_	7.32%	X_N,t−3_	0.65%
X_E,t−2_	3.84%	X_K,t−3_	0.65%
X_S,t−2_	3.26%	X_S,t−4_	0.64%
X_N,t−2_	2.79%	X_N,t−4_	0.57%
X_C,t−2_	1.89%	X_E,t−3_	0.53%
X_K,t−1_	1.33%	X_E,t−4_	0.52%
X_T,t−3_	0.92%	X_K,t−4_	0.52%
		Total	100.00%
